# Comprehensive first–trimester targeted metabolomics for early prediction and understanding of GDM pathophysiology

**DOI:** 10.3389/fmolb.2026.1760710

**Published:** 2026-02-16

**Authors:** Patrycja Mojsak, Adrian Godlewski, Krzysztof Sołowiej, Agieszka Kulczynska–Przybik, Sandra Chmielewska, Barbara Mroczko, Małgorzata Szelachowska, Adam Krętowski, Michał Ciborowski

**Affiliations:** 1 Metabolomics and Proteomics Laboratory, Clinical Research Centre, Medical University of Bialystok, Bialystok, Poland; 2 Department of Neurodegeneration Diagnostics, Medical University of Bialystok, Bialystok, Poland; 3 Department of Endocrinology, Diabetology and Internal Medicine, Medical University of Bialystok, Bialystok, Poland; 4 Department of Medical Biochemistry, Medical University of Bialystok, Bialystok, Poland

**Keywords:** GDM, targeted analysis, LC–MS/MS, plasma, diagnostic panel, pathophysiology

## Abstract

**Introduction:**

Gestational diabetes mellitus (GDM) is among the most common metabolic disorders during pregnancy, and early detection is key to reducing complications for both mother and child. Mass spectrometry–based metabolomics enables detailed metabolite profiling, offering opportunities not only for early diagnosis and risk prediction but also for understanding the pathophysiological mechanisms that drive the development of GDM.

**Methods:**

For the first time, an analysis of such a large number of metabolites was conducted: over 1,000 metabolites across 39 biochemical classes, including 912 lipids and 107 small molecules, were measured in first-trimester plasma from women with abnormal or normal fasting plasma glucose who later developed GDM, as well as from controls with normal glucose tolerance. Statistical analyses included Kruskal–Wallis ANOVA with Conover–Iman post hoc tests, Wilcoxon signed-rank tests for longitudinal changes, and ROC analysis to assess predictive and diagnostic performance. Spearman’s rank correlations were used to examine relationships between metabolites and clinical parameters.

**Results:**

Distinct metabolic signatures in the first trimester were associated with later GDM development. A prognostic panel, including TG (18:1_36:6), Hex2Cer(d18:1/14:0), valine, PS(36:1), TG (17:2_36:3), p-cresol sulfate, and PC(O–42:4), accurately predicted GDM (AUC = 0.934). A diagnostic panel comprising PE (P–18:0/22:4), glycine-conjugated cholic acid, LPC (20:3), carnitine esters, and arginine detected early signs of carbohydrate metabolism issues (AUC = 0.821). Women with normal fasting glucose who later developed GDM exhibited significant lipid alterations, whereas those with early fasting irregularities showed a partially GDM-like profile. Correlation analyses revealed distinct inflammatory and hormonal networks, with TNF–α–induced lipid remodelling linked to early dysglycaemia.

**Conclusion:**

First-trimester metabolomic signatures hold significant promise for early prediction, diagnosis, and understanding of GDM, enabling personalised risk assessment and timely intervention during pregnancy.

## Introduction

The global increase in obesity and maternal age has significantly contributed to the increasing prevalence of gestational diabetes mellitus (GDM) (Choudhury and Devi Rajeswari, 2021; Alesi et al., 2021). GDM currently affects about 14% of pregnancies, although prevalence estimates vary depending on population characteristics and diagnostic criteria (Eades et al., 2024). In clinical practice, GDM is most commonly diagnosed between 24 and 28 weeks of gestation, typically using glucose challenge tests (GCTs) or oral glucose tolerance tests (OGTTs). Although there is widespread awareness of the harmful effects of maternal hyperglycemia, there remains no consensus on the best methods for screening, testing, and diagnosing GDM, as shown by the more than 30 guidelines available worldwide (Mahmoud et al., 2024). Differences encompass diagnostic thresholds, screening methods, testing techniques, and the timing of diagnosis.

However, increasing evidence suggests that the metabolic disturbances underlying GDM may develop well before symptoms appear, possibly during the first trimester or even in the preconception period (Zhu et al., 2022). These findings emphasise the importance of developing more accurate tools for earlier detection and timely intervention. Early diagnosis of GDM is crucial, as untreated maternal hyperglycemia raises the risk of complications such as preeclampsia, macrosomia, shoulder dystocia, neonatal hypoglycemia, and a long–term predisposition to type 2 diabetes mellitus (T2DM) in both mother and child (Buchanan et al., 2012). Despite advancements in obstetric care, the pathophysiology of GDM remains complex, driven by insulin resistance, chronic low–grade inflammation, altered adipokine signalling, placental dysfunction, and genetic predisposition (Plows et al., 2018). The first trimester offers a particularly critical window for prediction and prevention, as it precedes the progressive insulin resistance that develops later in pregnancy. However, most studies are limited by small sample sizes or retrospective designs (Mahmoud et al., 2024). Furthermore, traditional glycemic markers such as fasting glucose, glycated haemoglobin (HbA1c), and fructosamine offer only moderate predictive value and fail to capture the full complexity of early metabolic changes (Mittal et al., 2025).

Metabolomics has emerged as a promising approach to address these gaps. By enabling high–throughput analysis of small molecules in biological samples, metabolomics provides an integrated view of overall metabolic activity (Qiu et al., 2023). Plasma and serum, although obtained invasively, are commonly used samples because of their availability and high metabolite content. They reflect biochemical changes across multiple organs and have already shown potential for identifying biomarkers for metabolic disorders such as T2DM, metabolic syndrome, and GDM (Jung et al., 2025; Wang Q. Y. et al., 2021). Both targeted and untargeted metabolomic approaches have been utilised in GDM research, resulting in the identification of metabolites with diagnostic or prognostic potential (Xu et al., 2025; Burzynska-Pedziwiatr et al., 2022; Donovan et al., 2018). However, to date, no study has systematically applied a targeted approach to such a wide range of metabolites during early pregnancy. Combining metabolomic profiling with clinical variables, such as pre–pregnancy body mass index (BMI), maternal age, blood pressure, and placental function markers, may further enhance predictive accuracy. Nonetheless, results across populations and analytical platforms remain inconsistent, emphasising the need for prospective studies with well–defined cohorts (Alesi et al., 2021; Roverso et al., 2023).

This study aims to prospectively assess first–trimester plasma metabolomic signatures in pregnant women who later develop GDM, compared with controls with normal glucose tolerance. For the first time, we used a targeted approach to examine over 1,000 metabolites across 39 biochemical classes, including 107 small molecules and 912 lipids, to develop a prognostic panel that predicts second–trimester GDM risk from first–trimester data, as well as a diagnostic panel that identifies early disruptions in carbohydrate metabolism. Our objective was to identify sensitive and specific biomarkers for earlier risk stratification and diagnosis, ultimately enabling timely interventions to improve maternal and fetal outcomes. Finally, to better understand the pathophysiological mechanisms behind GDM, we conducted correlation analyses among the identified metabolites, metabolic pathways, and clinical parameters in both study groups.

## Materials and methods

### Study groups

**FIGURE 1:**
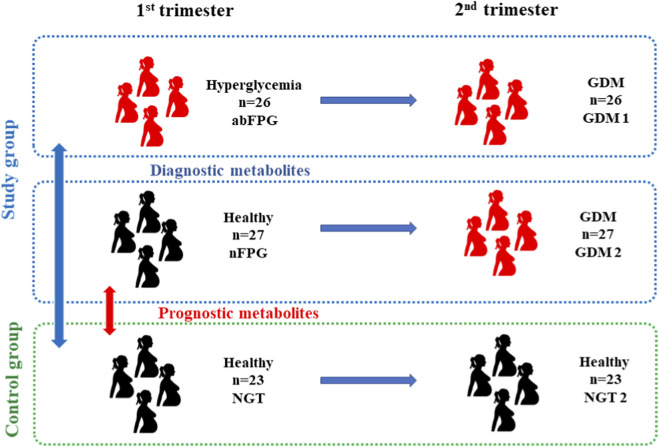
Schematic representation of the study and control groups.

A total of 662 pregnant women were screened for GDM at the Department of Endocrinology, Diabetology, and Internal Medicine (Medical University of Bialystok, Poland). We analyzed samples from pregnant women with impaired carbohydrate metabolism defined as abnormal fasting plasma glucose (abFPG; n = 26), or with normoglycemia (nFPG; n = 27) in the first trimester who subsequently developed GDM in the second trimester (GDM 1 and GDM 2), and from a control group of women with normal glucose tolerance (NGT; n = 23) who did not develop GDM in the second trimester (NGT 2) (Figure 1). Participants were selected to ensure comparability across groups with respect to potential confounding factors, including pre-pregnancy BMI, weight change during pregnancy, medication use, diet, and physical activity, based on information collected through structured questionnaires. GDM was diagnosed according to the IADPSG criteria (Lee et al., 2020) if at least one of the following thresholds was met: fasting glucose ≥92 mg/dL, 1–hour glucose ≥180 mg/dL, or 2–hour glucose ≥153 mg/dL (Holman et al., 2008; Metzger et al., 2010). Controls (NGT) had fasting glucose <92 mg/dL, 1–hour glucose <180 mg/dL, and 2–hour glucose <153 mg/dL. All women had normal HbA1c levels <5.7% 17, 18. Descriptive characteristics and biochemical parameters of the study population are included in Table 1.

**TABLE 1:** Descriptive characteristics and biochemical parameters of the study population.

Parameters	abFPG (IT)	nFPG (IT)	NGT (IT)
n	26	27	23
Age	32.8 (28.0–38.5)	29.8 (26.0–32.0)	32.2 (27.5–32.5)
Weight before pregnancy	70.6 (61.0–78.5)	64.9 (58.5–70.0)	65 (59.0–72.5)
BMI (kg/m2)	25.2 (21.7–28.0)	23.9 (21.2–26.0)	23.1 (20.7–24.9)
Total cholesterol	169.6 (154.8–191.8)	170.0 (152.5–191.0)	169.7 (154.5–184.0)
LDL cholesterol	78.9 (62.0–98.6)	78.8 (64.6–95.8)	73.9 (62.2–84.9)
Triglycerides	99.7 (79.3–121.0)	95.3 (64.5–125.5)	85.8 (56.0–107.0)
Glucose 0' (mg/dL) (1T)	96.5 (94.0–98.0)	83.3 (82.0–85.0)	88.9 (85.0–90.0)
Glucose 1 h (mg/dL) (1T)	147.6 (141.0–156.0)	107.1 (99.6–132.50	–
Glucose 2 h (mg/dL) (1T)	147.6 (141.0–156.0)	124.2 (114.5–156.5)	–
Insulin 0' (µIU/mL) (1T)	11.7 (9.6–13.2)	13.6 (9.3–16.5)	–
HOMA–IR	121.3 (91.3–151.6)	148.0 (111.1–187.0)	120.2 (47.7–166.5)
HOMA %β	2.6 (1.8–2.4)	2.2 (2.0–2.9)	1.6 (0.6–2.0)
Hb1Ac	5.3 (5.0–5.5)	5.1 (4.9–5.4)	5.1 (4.9–5.3)
IL–6	4.95 (4.03–6.24)	3.72 (3.33–4.03)	3.54 (3.20–4.30)
Leptine	5844 (4082–9376)	4685.5 (3612.25–7830)	4617 (3482–7152.5)
PYY	137.02 (130.17–145.06)	130.17 (124.36–139.33)	122.69 (113.81–128.60)
TNF alpha	7.87 (6.91–8.95)	7.53 (6.15–9.03)	7.39 (5.96–8.77)
Glucose 0'(mg/dL) (2T)	96 (93.5–97.0)	94.3 (93.0–96.0)	82.4 (75.0–88.0)
Glucose 1 h (mg/dL) (2T)	158.2 (146.2–165.0)	144.9 (109.0–175.0)	–
Glucose 2 h (mg/dL) (2T)	164.6 (153.5–182.0)	117.2 (96–144.8)	–

### Targeted analysis

**FIGURE 2:**
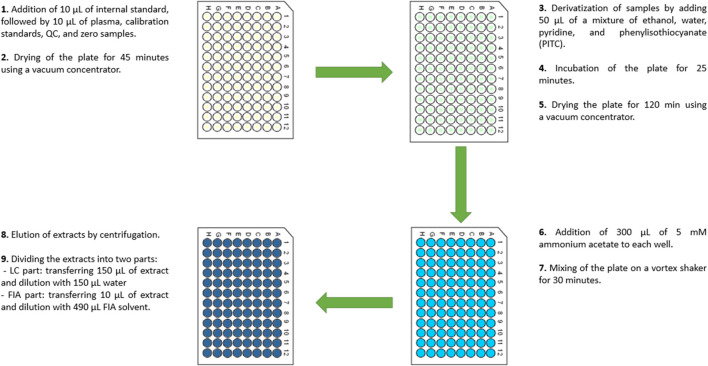
Scheme of plasma sample preparation using a commercially available kit.

Plasma samples were prepared using a commercially available kit (MxP® Quant 500 XL) that measures 1,019 metabolites, including 107 small molecules and 912 lipids, following the manufacturer’s protocol. Shortly before or during the analyses, the samples were thawed on ice. Each of the two reaction plates was loaded with 10 μL of quality control samples, calibration standards, blank samples, and test samples (Figure 2). Samples were evaporated in a vacuum concentrator. The metabolites on the first plate were then derivatized for polar compound analysis using a mixture of ethanol, water, pyridine, and phenyl isothiocyanate, followed by a 1-h incubation. The reaction plate was subsequently evaporated again. In the next step, the metabolites from the filters of both reaction plates were extracted using 5 mM ammonium acetate. For polar compound analysis, 150 µL of the extract was diluted with an equal volume of water. For lipid analysis, the extract was diluted 1:49 with methanol containing the mobile phase modifier provided in the kit. Additionally, lipids from the second plate (not derivatized) were prepared by diluting 50 µL of the extract with 450 µL of methanol containing the same mobile phase modifier. Metabolites in the prepared extracts were measured using an ultra–high–performance liquid chromatograph (Nexera LC–40, Shimadzu) coupled with a tandem mass spectrometer (QTrap 6,500+, Sciex) operating in both positive and negative modes in multiple reaction monitoring. Metabolite identification was performed using a targeted approach based on predefined multiple reaction monitoring transitions, retention times, and internal standards provided within the MxP® Quant 500 XL kit, according to the manufacturer’s validated protocol. Raw spectral data were processed in WebIDQ software (Biocrates Life Science AG) for signal integration, concentration estimation, and normalization.

Initial data quality control assessment was performed using Sciex OS software (ver. 3.1.6.44, DH Technologies Development Pte Ltd). Spectral data integration, quantification, and normalisation were carried out in WebIDQ (ver. DB123–758, Biocrates, Life Science AG). Data normalization was conducted based on repeated QC samples following the manufacturer’s guidelines. The data matrix was filtered to retain metabolites with reliable quantification.

### Measurement of cytokines and hormones

Plasma concentrations of leptin, interleukin–6 (IL–6), tumour necrosis factor alpha (TNF–α), and peptide YY (PYY) were measured using a Multiplex assay (Merck, Germany) according to the manufacturer’s instructions. The analyses were performed on a Luminex–based platform, and the results are presented in Table 1.

### Statistical analysis

Multivariate statistical methods were used to evaluate data quality. Principal component analysis (PCA) was applied to assess the clustering of quality control (QC) samples. Group separation in the first trimester was visualised using partial least squares discriminant analysis (PLS–DA) in SIMCA−P+ 13.0.3.0 software (Umetrics, Umeå, Sweden). To identify metabolites that differed among the three groups (abFPG, nFPG, and NGT), a non–parametric Kruskal–Wallis ANOVA was performed on the first–trimester data. Significant differences were further examined using the Conover–Iman *post hoc* test for pairwise comparisons (p < 0.05). Additionally, the Wilcoxon signed–rank test was used to assess differences between the first and second trimesters within patients. All statistical analyses were conducted in R (version 4.0.0; https://www.R–project.org/, accessed on 15 February 2025). Receiver operating characteristic (ROC) curve analysis of significant metabolites was conducted using MetaboAnalyst 5.0. Validation of the OPLS–DA models was carried out by cross–validation using the leave–1/3–out approach as described previously (Ciborowski et al., 2012). The dataset was divided into three subsets: one-third of the samples were excluded, and the model was built using the remaining two-thirds. The model then predicted the excluded samples. This procedure was repeated until each sample had been predicted at least once, and the percentage of correctly classified samples was calculated for each iteration. Lastly, Spearman’s rank correlation analysis (in R) was employed to explore associations between metabolomic data and clinical characteristics (version 4.5.1).

## Results

### Metabolomics

The study included women with abFPG and nFPG in the first trimester who developed GDM in the second trimester, as well as NGT controls who did not develop GDM. The study groups were well matched for age, body weight, and BMI, with no statistically significant differences among them (p > 0.05), thereby reducing the risk of confounding by these anthropometric variables (see Table 1). PLS–DA revealed group separation in metabolite profiles between groups (Supplementary Figure S1), with score plots showing discrimination between groups and model performance of *R* ^2^ = 0.671 and Q^2^ = 0.494; *p*–value = 0.007.

First, we compared patient profiles across trimesters, namely, abFPG vs. GDM 1, nFPG vs. GDM 2, and NGT vs. NGT 2. The Wilcoxon test results summarized in Supplementary (Supplementary Table S1) showed statistical significance for 10 metabolites (phosphatidylglycerols, n = 4; sphingomyelins, n = 2; others, n = 4) and 183 metabolites (Supplementary Table S1) (mainly triacylglycerols, n = 108; phosphatidylcholines, n = 24; phosphatidylglycerols, n = 13; others, n = 38) in the abFPG vs. GDM 1 and nFPG vs. GDM 2 comparisons, respectively. The complete list of significantly altered metabolites is provided in Supplementary Table S2. Comparing healthy patient profiles allowed identification of metabolites whose changes were influenced by time.

## Results for the first trimester of pregnancy

Our analysis identified 34 metabolites with statistically significant differences between the nFPG and NGT groups in the first trimester. These metabolites were mainly glycerolipids, followed by amino acids and glycerophospholipids (Table 2). PLS–DA score plots (Supplementary Figure S2) showed clear separation between the nFPG and NGT groups, with *R* ^2^ (cum) = 0.658 and Q^2^ (cum) = 0.512 (*p*–value = 0.025). For the nFPG vs. NGT comparison, 1/3rd (8 samples) were excluded in each iteration and predicted in 12 repetitions until all samples had been predicted at least once. The percentage of correctly classified samples was calculated as 81.4% ± 18.6%. Among the significant metabolites, amino acids valine (FC = 1.22, pcorr = −0.43, VIP = 1.08), isoleucine (FC = 1.17, pcorr = −0.42, VIP = 1.12), and Hex2Cer d18:1/14:0 (FC = 1.32, pcorr = −0.42, VIP = 2.10) were elevated, whereas di–and tri–acylglycerols (FC = 0.58–0.80) were decreased in nFPG compared with NGT.

**TABLE 2:** Statistically significant changes in plasma metabolites between nFPG and NGT.

Subclass	Metabolites	CV in QCs	FC	Pcorr	VIP	*p*–value
Amino acids, peptides, and analogues	Arg	5.4	0.88	0.42	1.31	<0.05
Amino acids, peptides, and analogues	Val	4.9	1.22	−0.43	1.08	<0.05
Amino acids, peptides, and analogues	Ile	5.9	1.17	−0.42	1.12	<0.05
Amino acids, peptides, and analogues	Cit	6.1	0.83	-	-	3.89E-02
Triacylglycerols	TG 17:2_36:3	25.3	0.58	0.40	2.79	<0.05
Triacylglycerols	TG 18:3_36:4	6.3	0.65	0.55	3.29	<0.05
Triacylglycerols	TG 18:2_36:4	6.8	0.66	0.46	2.64	<0.05
Triacylglycerols	TG 18:1_36:5	5.1	0.67	0.55	3.08	<0.05
Triacylglycerols	TG 18:3_36:3	5.6	0.67	0.54	3.15	<0.05
Triacylglycerols	TG 18:2_36:5	5.2	0.67	0.52	2.92	<0.05
Triacylglycerols	TG 18:3_34:3	12.0	0.67	0.48	2.72	<0.05
Triacylglycerols	TG 18:2_36:3	6.6	0.68	0.43	2.42	<0.05
Triacylglycerols	TG 18:1_36:4	7.9	0.69	0.49	2.75	<0.05
Triacylglycerols	TG 18:2_34:3	8.6	0.69	0.41	2.20	<0.05
Triacylglycerols	TG 22:1_32:5	26.7	0.69	0.52	3.73	<0.05
Triacylglycerols	TG 18:1_36:6	13.7	0.51	0.54	3.18	<0.05
Triacylglycerols	TG 18:2_34:4	7.7	0.70	0.43	2.34	<0.05
Triacylglycerols	TG 18:3_36:2	7.0	0.70	0.52	2.93	<0.05
Triacylglycerols	TG 18:1_36:3	5.7	0.71	0.43	2.36	<0.05
Triacylglycerols	TG 18:3_34:2	7.1	0.71	0.41	2.22	<0.05
Triacylglycerols	TG 18:0_36:5	6.6	0.71	0.40	2.06	<0.05
Triacylglycerols	TG 18:2_36:2	7.2	0.72	0.42	2.25	<0.05
Triacylglycerols	Cer d18:2/24:0	8.0	0.76	0.46	1.98	<0.05
Triacylglycerols	Cer d18:2/22:0	9.9	0.80	0.42	1.81	<0.05
Diacylglycerols	DG 18:2_18:2	9.5	0.83	0.40	1.81	<0.05
Glycerophosphocholines	PC O–42:4	5.9	1.17	−0.36	1.26	<0.05
Glycerophosphoserines	PS 36:1	17.5	1.38	−0.42	2.10	<0.05
Cresols	p–Cresol–SO4	4.7	0.74	-	-	1.06E-02
Carbohydrates and carbohydrate conjugates	Hexose	6.3	0.82	0.48	1.24	<0.05
Ceramides	Cer d18:2/20:0	11.3	0.79	0.35	1.57	<0.05
Others	Hex2Cer d18:1/14:0	9.7	1.32	−0.44	1.93	<0.05
Metabolic proess	p–Cresol–SO4 synthesis	4.0	0.75	-	-	2.02E-02
Metabolic process	TMAO synthesis (direct)	5.9	0.85	-	-	2.87E-02
Metabolic process	Ratio of Hex2Cer to cer	6.4	1.13	-	-	3.28E-02

**FIGURE 3:**
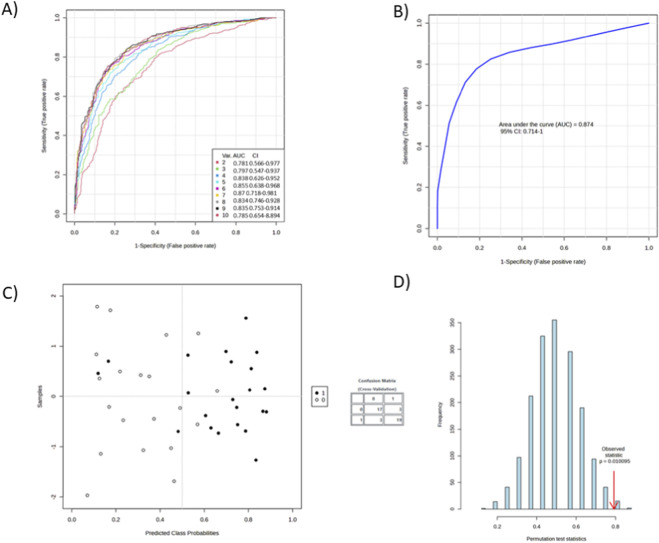
Performance of SVM-based classification models for distinguishing study groups using metabolomic data. **(A)** Receiver Operating Characteristic (ROC) curves for ten SVM models constructed using different variable sets. Area under the curve (AUC) values with 95% confidence intervals indicate strong discriminative ability across models (AUC range: 0.78–0.88). **(B)** ROC curve for the final optimized SVM model showing an AUC of 0.874 (95% CI: 0.71–0.94), confirming robust predictive performance. **(C)** Cross-validated sample plot showinging predicted class probabilities for the two groups (black and white circles). The inset confusion matrix summarizes classification outcomes, indicating high accuracy of group discrimination. **(D)** Results of the permutation test assessing the statistical significance of the model. The distribution of permuted test statistics (blue bars) is compared with the observed statistic (red arrow). The low permutation *p*–value (p = 0.001095) confirms that the observed model performance is not due to chance.

**FIGURE 4:**
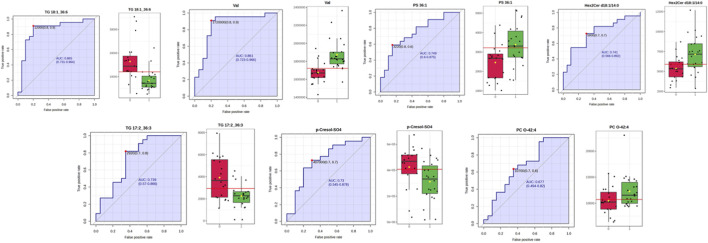
ROC curves and box plots for selected prognostic metabolites.

Significant metabolites were evaluated using ROC analysis. Using support vector machine (SVM) modelling, the top 10 metabolites were identified based on relative abundance across groups. Among these, seven showed the highest discriminatory power, and individual ROC curves were generated (Figures 3, 4).

Next, we compared the metabolic profiles of patients with abFPG and NGT. Based on both univariate and multivariate analyses, 25 metabolites were significantly different (Table 3). These included phosphatidylcholines (11 metabolites), acylcarnitines (3 metabolites), along with cholesterol esters, diacylglycerols, and amino acids. In the PLS–DA score plots (Supplementary Figure S3), the abFPG and NGT groups were clearly separated, with R2 (cum) = 0.623 and Q2 (cum) = 0.436 (p = 0.045). For the abFPG vs. NGT comparison, 1/3rd (9 samples) were excluded in each iteration and predicted in 10 repetitions until all samples had been predicted at least once. The percentage of correctly classified samples was calculated as 91.4% ± 8.6%. Among the statistically significant metabolites, LPC 20:3 (FC = 1.11, pcorr = −0.40, VIP = 1.17), PE (P–18:0/22:4) (FC = 1.19, pcorr = −0.40, VIP = 1.66), and glycine–conjugated cholic acid (CA) (FC = 1.58, pcorr = −0.43, VIP = 4.33) were elevated.

**TABLE 3:** Statistically significant changes in plasma metabolites between abFPG and NGT.

Subclass	Metabolite	Pcorr	VIP	CV QC 2	FC	*p*–value
Acylcarnitines	C14:1	0.408354	1.88439	4.65	0.79	<0.05
Acylcarnitines	Carnitine esterification	−0.44316	1.54082	4.33	118.90	2.29E-02
Acylcarnitines	b-Oxidation	0.401895	1.37156	4.54	0.85	4.97E-02
Aminoacids	Arg	0.402644	1.25739	5.41	1.17	<0.05
Aminoacids related	AABA	0.385393	1.66262	5.85	0.88	<0.05
Aminoacids, cresols	p-Cresol-SO4 synthesis	0.40294	2.13178	4.04	0.72	5.37E-03
Bile acids	Gly conjugation of CA	−0.43167	4.33839	1.99	1.58	<0.05
Cholesterol esters	CE 22:6	0.520678	2.24273	12.68	0.76	<0.05
Cholesterol esters	CE 20:5	0.545206	2.2422	11.72	0.83	<0.05
Diacylglycerols	DG 18:2_18:2	0.395666	2.07818	9.51	0.81	<0.05
Diacylglycerols	DG 18:1_18:2	0.476113	1.75328	7.17	0.82	<0.05
Fatty acids	FA 20:1	0.423697	1.67932	12.39	0.87	<0.05
Phosphatidylcholines	PC 42:6	0.459186	1.74183	8.36	0.84	<0.05
Phosphatidylcholines	PC 42:5	0.41465	1.65591	5.45	0.85	<0.05
Phosphatidylcholines	PC 38:0	0.476919	1.58546	7.18	0.86	<0.05
Phosphatidylcholines	PC 38:6	0.500935	1.74777	4.97	0.86	<0.05
Phosphatidylcholines	PC 40:6	0.479911	1.58007	5.17	0.87	<0.05
Phosphatidylcholines	PC 40:3	0.403	1.43579	6.33	0.89	<0.05
Phosphatidylcholines	PC O–40:6	0.477937	1.54401	3.94	0.90	<0.05
Phosphatidylcholines	PC 36:5	0.442072	1.81713	5.79	0.91	<0.05
Phosphatidylcholines	PC O–38:0	0.501355	1.69728	3.77	0.91	<0.05
Phosphatidylcholines	PC 40:2	0.402605	1.18564	6.26	0.92	<0.05
Phosphatidylcholines	LPC 20:3	−0.4029	1.17791	7.31	1.11	<0.05
Phosphatidylethanolamines	PE P–18:0/22:4	−0.40081	1.66842	5.00	1.20	<0.05
Phosphatidylglycerols	PG 22:6_22:6	0.472434	1.48358	6.74	0.89	<0.05

**FIGURE 5:**
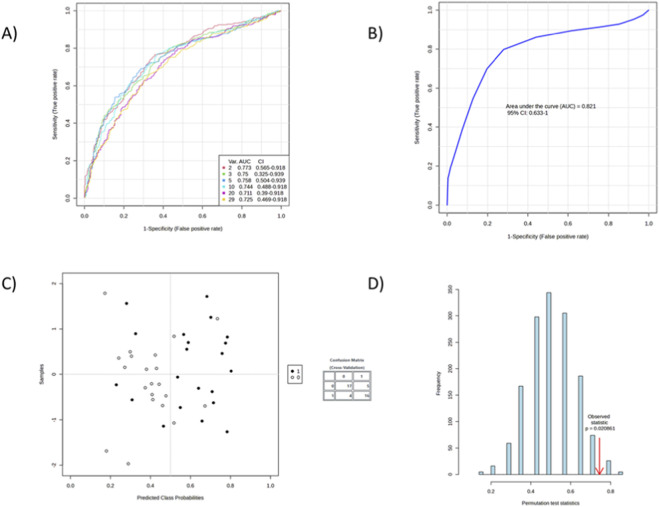
Support Vector Machine (SVM) modeling performance for classification based on metabolomic data. **(A)** Receiver Operating Characteristic (ROC) curves showing the classification performance of six SVM models built using different subsets of metabolites. The area under the curve (AUC) values with corresponding 95% confidence intervals demonstrate good discriminative ability across all models (AUC range: 0.77–0.82). **(B)** ROC curve for the final optimized SVM model, yielding an AUC of 0.821 (95% CI: 0.63–1.0), indicating strong predictive accuracy. **(C)** Cross-validation results displayed as a sample plot of predicted class probabilities for the two groups (black and white circles). The inset confusion matrix summarises classification accuracy and shows a clear separation between classes. **(D)** Permutation test assessing the statistical significance of the model. The distribution of permuted test statistics (blue bars) is compared with the observed statistic (red arrow). The low permutation *p*–value (p = 0.002061) confirms that the model performance is significantly better than expected by chance.

**FIGURE 6:**
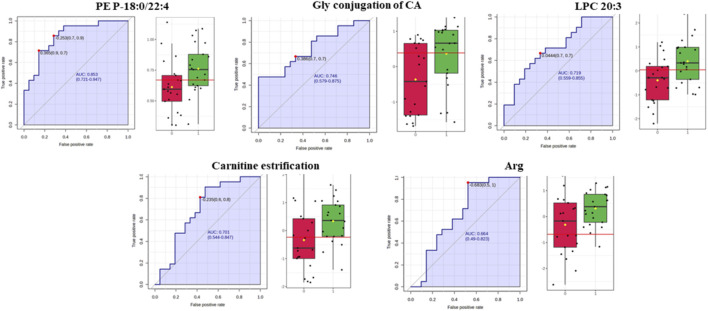
ROC curves and box plots for selected diagnostic metabolites.

ROC curve analysis was performed for significant metabolites to identify potential diagnostic biomarkers. Based on the analysis conducted in MetaboAnalyst 5.0, seven metabolites with the highest discriminatory power (Figure 5) were selected, and individual ROC curves were constructed for each (Figure 6).

### Correlation between metabolomics and clinical data

A correlation analysis was performed between clinical data (peptide YY (PYY), interleukin–6 (IL–6), High–Density Lipoprotein (HDL), Homeostatic Model Assessment of Insulin Resistance (HOMA–IR), Homeostatic Model Assessment of β–cell function (HOMA–beta), Tumour Necrosis Factor alpha (TNF alpha) and metabolites (AAs–amino acids, ceramides (Cers), Phosphatidylcholines (PCs), Phosphatidylethanolamines (PEs), Phosphatidylglycerols (PGs), considering their grouping. All correlations are shown in Supplementary Figure S4.

#### Amino acids

In the nFPG group (see Supplementary Figure S4a), IL–6 showed a positive correlation with amino acids such as Trp betaine, probetaine, and AABA, and a negative correlation with hCys. PYY was positively correlated with T4–OH–Pro, whereas TNF–α was negatively correlated with Phe, Arg, and ALA, but positively correlated with Trp betaine. Additionally, HOMA–β was negatively correlated with Cys, whereas HbA1c was positively correlated. In the abFPG group, IL–6 was positively correlated with serine, and PYY was negatively correlated with tryptophan. HOMA–β was negatively correlated with creatinine.

### Ceramides

In the nFPG group (Supplementary Figure S4b), IL-6 was negatively associated with Cer d18:1/26:1, whereas leptin and PYY showed multiple positive correlations with several ceramide species. In contrast, in the abFPG group, only TNF-α showed positive correlations with selected ceramides, and fasting glucose correlated positively with Cer d18:2/24:1.

### Glycosylceramides

In the nFPG group, leptin, TNF-α and PYY showed multiple positive correlations with various glycosylceramides (Supplementary Figure S4c), including several HexCer and Hex2/3Cer species. PYY, in particular, was strongly associated with a broader range of complex glycosylceramides. In contrast, in the abFPG group, leptin showed no significant associations, PYY correlated with only a few species, and TNF-α displayed positive correlations with several selected glycosylceramides.

#### Phosphatidylcholines (PCs)

In the nFPG group, phosphatidylcholines (PCs) were positively correlated with leptin, PYY, total cholesterol, and HDL. Conversely, in the abFPG group, these associations were not observed; instead, numerous positive correlations between TNF–α and various PCs were observed. Additionally, HbA1c showed a positive correlation with PC 40:4 and negative correlations with PC O–36:2, PC O–34:3, PC O–34:2, and PC O–28:2 (see Supplementary Figure S4d).

#### Phosphatidylethanolamines (PEs)

In the nFPG group (see Supplementary Figure S4e), several phosphatidylethanolamines (PEs) were positively associated with leptin, PYY, total cholesterol, and HDL. In the abFPG group, correlations were more heterogeneous: some PEs correlated positively with leptin, whereas others showed negative associations, and TNF-α was positively related to a few PEs. HbA1c was positively associated with LPE 18:0 and LPE 16:0 and negatively with PE-P 18:0/18:2 and PE 44:6, suggesting selective remodeling of PE species with glycaemic status.

#### Phosphatidylglycerols (PGs)

In the nFPG group, leptin showed positive correlations with two phosphatidylglycerol (PG) species, while PYY correlated positively with 18 PG species. Positive associations of these PGs with HOMA–IR, HOMA–BETA, and HDL were also observed. In the abFPG group, the correlation pattern was more diverse. Negative correlations were observed between IL–6 and PG 18:2/18:3, and between leptin and PG 20:4_22:1, indicating impaired hormonal regulation. Conversely, TNF–α showed positive correlations with PG 18:2_22:1 and PG 18:2_20:0. Additionally, several metabolites outside the PG class correlated negatively with HbA1c.

#### Phosphatidylinositols

In the nFPG group (see Supplementary Figure S4g), leptin showed positive correlations with four metabolites, whereas PS 36:1 showed a negative correlation. Several metabolites also showed positive correlations with PYY. HOMA–IR was negatively correlated with PS 36:1, whereas HOMA–beta showed positive correlations with PS 36:7 and SPBP d17:1. In the abFPG group, no significant correlations were observed between leptin or PYY and metabolites. HOMA–IR was negatively correlated with PS 36:1, and HOMA–beta showed negative correlations with PS 36:1 and positive correlations with PS 36:7 and SPBP d17:1. Additionally, TNF–α was positively correlated with PS 36:5, and HbA1c showed strong negative correlations with PI 16:1/18:2 and PI 14:0/18:2.

### Metabolic process

In the nFPG group, IL-6 was positively associated with indices of acylcarnitine metabolism, phospholipase activity, cortisone synthesis, and β-oxidation, and negatively associated with homocysteine synthesis. Leptin and PYY showed positive correlations with several phospholipid and sphingolipid pathways, including PUFA-containing species and glycosylceramides, whereas TNF-α was linked to higher PKU marker levels and lower lipopolysaccharide levels. HOMA-IR and HOMA-β were mainly related to altered acylcarnitine ratios and plasmalogen remodeling, while HbA1c was associated with markers of SCAD deficiency and cysteine synthesis, together with reduced proportions of unsaturated plasmalogens.

In the abFPG group, IL-6 was inversely related to phosphatidylserine synthase activity, whereas PYY showed broad negative associations with PUFA- and LPE-related pathways. TNF-α was positively associated with CACT deficiency, and HOMA-IR reflected reductions in monoacylglycerols and increases in LPE species. HOMA-β was positively associated with selected sphingomyelin and phosphatidylinositol species (see Supplementary Figure S4h).

## Discussion

Emerging metabolomics techniques have facilitated the identification of early biochemical changes associated with GDM, offering opportunities for improved risk prediction and disease stratification. For the first time, using a targeted analysis of over 1,000 metabolites across 39 biochemical classes (107 small molecules and 912 lipids) with a commercially available analytical kit, we developed a prognostic panel that predicts GDM risk in the second trimester from first–trimester measurements. Additionally, we established a diagnostic panel that detects disturbances in carbohydrate metabolism as early as the first trimester, which may lead to GDM in the second trimester. While our study focuses on metabolomic profiling, previous research has demonstrated the utility of ensemble-based prediction models and explainable artificial intelligence techniques for forecasting GDM risk (Hassan et al., 2025a) and preterm birth (Hassan et al., 2025b), as well as the potential of Internet of Things devices for remote monitoring of maternal and fetal health (Ahmad et al., 2025). Our longitudinal analysis of patient profiles further supports the rationale for these studies. We observed far fewer significant metabolite differences between patients with early carbohydrate metabolism disturbances who later developed GDM (abGDM vs. GDM 1) than between patients who were initially metabolically normal but developed GDM in the second trimester (nFPG vs. GDM 2). Specifically, 10 metabolites were significant in the abGDM versus GDM 1 comparison (mainly phosphatidylglycerols and sphingomyelins), whereas 183 metabolites were significant in the nFPG versus GDM 2 comparison (mainly triacylglycerols, phosphatidylcholines, and phosphatidylglycerols). These findings suggest that abGDM patients already exhibit a partially GDM–like metabolic profile in early pregnancy, while initially metabolically normal patients show more pronounced changes later. Natural temporal and pregnancy–related metabolic variations were controlled by comparing healthy women across trimesters. Overall, metabolomics can detect subclinical metabolic alterations before clinical GDM onset, as our findings confirm.

Furthermore, based on the correlation analyses (see Supplementary Figure S4), distinct patterns of associations between metabolites and clinical parameters were observed in the nFPG and abFPG groups. In the nFPG group, metabolites such as ceramides, glycosylceramides, and various phospholipids mainly correlated with leptin, IL–6, and PYY, but not with TNF–α. Conversely, in the abFPG group, correlations with IL–6, leptin, and PYY were absent, whereas significant associations with TNF–α were observed. These findings suggest that women with abFPG display a shift towards a more TNF–α–driven inflammatory profile, whereas those with nFPG show metabolite–adipokine associations indicative of low–grade, physiological inflammation typical of early normal pregnancy. Such divergent patterns may reflect distinct mechanisms of metabolic adaptation, with TNF–α–mediated lipid alterations potentially serving as early markers of insulin resistance. This interpretation aligns with evidence indicating that certain adipokines, such as visfatin, are associated with an increased risk of future diabetes, while others (including leptin, TNF–α, and IL–6) show variable links depending on metabolic status (Sun et al., 2023).

### Early predictive metabolomic signatures of gestational diabetes mellitus in the first trimester

The metabolomic differences observed in women with nFPG compared with those with NGT suggest that disruptions in key metabolic pathways may already be present in early pregnancy. Elevated levels of branched–chain amino acids (BCAAs) (e.g., valine, isoleucine) and reduced levels of other amino acids (e.g., arginine, citrulline) are consistent with reports linking altered amino acid profiles to metabolic dysfunctions in GDM (Wang et al., 2025). Several studies have suggested that BCAAs and aromatic amino acids (AAAs) are implicated in the onset, progression, and remission of insulin resistance, type 2 diabetes, and obesity (Tulipani et al., 2016; Wang et al., 2011). A cohort study in Chinese women showed notable differences in alanine, isoleucine, and tyrosine concentrations during early pregnancy between those who later developed GDM and those who remained normoglycemic (Jiang et al., 2020). We observed an increase in specific ceramide: Cer d18:2/20:0 (FC = 1.51) alongside a decrease in di–and triacylglycerols, indicating reprogramming of lipid metabolism, shifting the balance between fatty acid storage and utilisation. It has been confirmed that ceramides intensify endoplasmic reticulum stress promote pancreatic beta cell apoptosis and inhibit insulin signaling (Galadari et al., 2013). These findings align with evidence from earlier studies (Furse et al., 2019), which demonstrated that disturbances in phospholipid and triglyceride metabolism were already apparent at the start of the second trimester in obese women who later developed GDM. Notably, significant differences in lipid, sterol, and triglyceride species could be detected at least 10 weeks before diagnosis, highlighting early changes in oxidative and lipoprotein–related pathways. Overall, these observations suggest that alterations in lipid metabolism may serve as early markers of insulin resistance and contribute to the underlying pathophysiology of GDM, supporting their inclusion in advanced predictive models.

An optimized multi–metabolite panel (see Figure 6) including TG (18:1_36:6), Hex2Cer(d18:1/14:0), valine, PS(36:1), TG (17:2_36:3), p–Cresol–sulphate (SO_4_), and PC(O–42:4) emerged as particularly informative for early prediction of GDM. Importantly, many of these metabolites have been reported in previous studies, highlighting consistency in altered lipid and amino acid metabolism (Zhu et al., 2022; Jung et al., 2025; Elkanawati et al., 2024; Yaribeygi et al., 2020; Chen et al., 2018) while p–Cresol–SO_4_ represents a novel biomarker not widely described in the context of GDM. These metabolites represent diverse biological processes, spanning amino acid metabolism, lipid remodelling, bile acid conjugation, and inflammatory signalling. Similar metabolic interaction patterns, highlighting the interplay between lipid metabolism, inflammation, and insulin sensitivity, were also described in the study by Elkanawati et al. (Elkanawati et al., 2024), further supporting the concept that GDM development reflects a network of interrelated metabolic disturbances rather than a single defect (Plows et al., 2018).

Branched–chain amino acids (BCAAs), particularly valine, have been consistently associated with insulin resistance (Zhao et al., 2016). Elevated circulating BCAAs during early pregnancy are linked to impaired glucose metabolism and an increased risk of GDM (Lu et al., 2021; Razo-Azamar et al., 2023) and also contribute to the development of T2DM (Razo-Azamar et al., 2023). In our study, TNF–α was negatively correlated with phenylalanine, arginine, and alanine (see Supplementary Figure S4), suggesting that inflammatory pathways may interact with amino acid metabolism, potentially influencing insulin resistance and glucose homeostasis during pregnancy. These findings highlight the interplay between inflammation and amino acid dysregulation in the pathophysiology of GDM.

Hexosylceramides, such as Hex2Cer(d18:1/14:0), are glycosylated ceramide derivatives involved in cellular signaling, inflammation, and apoptosis (Zhang et al., 2024). Alterations in sphingolipid metabolism have been observed before GDM onset, highlighting their potential as early biomarkers. Lipidomic studies have further identified hexosylceramides (18:1/16:0, 18:1/18:0, 18:1/24:1) as first–trimester predictors of GDM (Zhang et al., 2024; Wang Y. et al., 2021). Including these sphingolipid species in early screening, alongside traditional risk factors, might improve the detection of women at high risk. Triacylglycerols such as TG (18:1_36:6) and TG (17:2_36:3) reflect broader disruptions in lipid metabolism. Elevated levels may indicate heightened hepatic *de novo* lipogenesis, impaired fatty acid oxidation, or lipotoxicity, all processes closely linked to insulin resistance (Zhang et al., 2024; Du et al., 2024; Wang et al., 2022). Similarly, changes in phospholipids, including PS(36:1) and PC(O–42:4), suggest disturbances in membrane composition and signaling pathways (Ventura et al., 2022; Bowman et al., 2021). In the nFPG group, leptin showed positive correlations with several glycosylceramides (see Supplementary Figure S4), while PYY was associated with more complex glycosylceramides. In contrast, in the abFPG group, leptin showed no significant associations, PYY correlated with only a few species, and TNF–α was strongly linked to multiple glycosylceramides. These findings suggest that specific hexosylceramides not only reflect early metabolic disruptions in GDM but also interact with hormonal and inflammatory pathways, potentially contributing to group–specific differences in disease progression consistent with observations reported by Yaribeygi et al. (2020).

A key finding of this study is p–Cresol–SO_4_, a gut microbiota–derived metabolite formed during the breakdown of AAAs such as tyrosine and phenylalanine. Elevated levels of this compound early in pregnancy may reflect altered gut microbial composition or increased colonic proteolytic fermentation, both of which are linked to metabolic issues (Flynn et al., 2025; Wu et al., 2025). p–Cresol–SO_4_ has been shown to affect host metabolism through various mechanisms: it can impair endothelial function, promote systemic and placental inflammation, and disrupt insulin signalling pathways, ultimately reducing insulin sensitivity and impairing glucose regulation, hallmarks of GDM pathophysiology (Koppe et al., 2013; Hasain et al., 2020). Additionally, p–Cresol–SO_4_ may act as a signalling molecule, influencing immune responses and metabolic flexibility, thereby increasing metabolic demands during pregnancy (Mittal et al., 2025). Its early detection in maternal plasma highlights the gut–microbiota axis as a potentially modifiable factor in GDM. These findings suggest that p–Cresol–SO_4_ and other identified metabolites could serve as potential therapeutic or intervention targets. Modulating sphingolipid or lipid metabolism, as well as gut microbiota composition, via dietary, probiotic, or pharmacological approaches may influence insulin sensitivity and inflammation, offering opportunities for early prevention of GDM. Based on Spearman correlation analysis (see Supplementary Figure S4), it was confirmed that p–Cresol–SO_4_ synthesis exhibited positive correlations with PYY. This suggests an interaction between the gut microbiota and hormonal signals regulating appetite and glucose metabolism in GDM. The early detection of p–Cresol–SO_4_ in maternal plasma highlights the gut–microbiota axis as a potentially modifiable factor in GDM development and supports the idea that dietary or probiotic interventions could aid in early risk stratification and prevention to conventional GDM testing.

### Early diagnostic metabolomic signatures of disruptions in glucose metabolism in the first trimester

Statistical analysis comparing abFPG with NGT revealed significant differences, particularly in PC and acylcarnitine metabolites. Altered PCs may indicate disruptions in membrane structure and lipid transport, potentially contributing to early insulin resistance (van der Veen et al., 2017). Based on correlation analysis, several PC metabolites also exhibited positive correlations with TNF–α, suggesting a link between PC alterations and pro–inflammatory pathways in early metabolic imbalance. Similarly, acylcarnitines, which are crucial for fatty acid transport and mitochondrial β–oxidation, may indicate impaired lipid oxidation and the build–up of lipotoxic intermediates (Visiedo et al., 2023). Altered acylcarnitine profiles also imply disruptions in carnitine esterification, a key process for fatty acid transport into mitochondria; disruptions here may lead to early mitochondrial dysfunction, lipid accumulation, and worsening insulin resistance (Xiang et al., 2025). These metabolite changes align with findings from an early prediction model in which short-chain acylcarnitines achieved a high discriminative performance (AUC = 0.934 (0.873–0.995)) for GDM detection in early pregnancy (Razo-Azamar et al., 2023).

Furthermore, our study identified an early diagnostic panel for carbohydrate metabolism disorders detectable in the first trimester, preceding the clinical onset of GDM. This panel included PE (P-18:0/22:4), glycine–conjugated cholic acid, LPC (20:3), carnitine esters, and arginine, with levels significantly higher in women who later developed GDM than in controls. Oxidative stress is an early factor in this metabolic process and is a recognized contributor to insulin resistance during pregnancy (Lappas et al., 2011). Elevated PE (P-18:0/22:4), a plasmalogen phospholipid, may indicate oxidative stress and membrane remodelling (Lappas et al., 2011). Plasmalogens act as both structural components of membranes and endogenous antioxidants, suggesting that their early accumulation reflects a compensatory response to oxidative imbalance. Similar early lipid alterations have been reported in large cohort studies, where metabolomic panels that included glycerophospholipids and bile acids achieved robust predictive performance for GDM (e.g., AUC = 0.85 for a model combining clinical factors and metabolites [10.1186/s12933-025-02978-0]. Although lipid alterations in GDM have been documented previously, these changes were mainly reported in later stages of gestation (Chen et al., 2018; Mao et al., 2017; McCabe and Perng, 2017). In contrast, our findings show that such lipid disturbances can be observed in the first trimester, well before clinical signs of GDM, highlighting their potential as early biomarkers for GDM risk assessment and prevention.

Alongside oxidative stress, bile acid metabolism appears to be disrupted. Elevated glycine–conjugated cholic acid observed in our study indicates an altered bile acid balance, a process increasingly recognized for its role in glucose metabolism and insulin sensitivity (Wang et al., 2024; Ferrell and Chiang, 2019). The accumulation of these bile acids may interfere with enterohepatic signalling, disrupting incretin secretion and hepatic glucose production, both of which are essential for maintaining glucose tolerance. Given their early occurrence, altered bile acid profiles may serve as actionable biomarkers and potential targets for dietary, probiotic, or pharmacological interventions aimed at improving glucose homeostasis and maternal metabolic stability, as gut microbiota–bile acid interactions have been implicated in modulating GDM risk. Importantly, these early changes could serve as potential biomarkers for identifying women at risk of developing GDM. This finding aligns with longitudinal studies reporting trimester–specific changes in total bile acids and their conjugation profiles, which are linked to progressive increases in insulin resistance indices (Gagnon et al., 2021; Zhu et al., 2019). Emerging evidence also indicates that disturbances in bile acid metabolism may interact with gut microbiota composition, further modulating maternal metabolic stability during pregnancy.

The role of inflammation is further emphasised by elevated LPC(20:3), a lysophosphatidylcholine implicated in endothelial dysfunction and low–grade inflammation (Hung et al., 2024). Increased LPC(20:3) levels may serve as an early indicator of pro–inflammatory signalling, potentially worsening insulin resistance and vascular dysfunction, aligning with previous studies linking lysophosphatidylcholines to GDM pathogenesis (Mittal et al., 2025). Similarly, higher arginine, a semi–essential amino acid and key precursor of nitric oxide (NO), may serve as a compensatory mechanism to maintain endothelial function. Elevated arginine levels (see Table 3) could support nitric oxide (NO) production, thereby mitigating endothelial dysfunction—a hallmark of GDM, and modulating insulin sensitivity through activation of the mechanistic target of rapamycin (mTOR) and AMP–activated protein kinase (AMPK) signalling pathways (Forzano et al., 2023; Zhang et al., 2025).

### Correlation of metabolic processes with clinical parameters

Spearman correlation analyses of metabolic processes with clinical parameters in the nFPG group revealed a coordinated network linking inflammatory markers, adipokines, gut hormones, and lipid and energy metabolism pathways. These associations suggest that subtle metabolic adaptations already present early in pregnancy could predispose individuals to impaired glucose regulation observed later in gestation. IL–6 showed positive correlations with ratios of short–chain to medium–and long–chain acylcarnitines, the acetylcarnitine–to–carnitine ratio, PLD and PLAS2 activity, cortisone synthesis, and β–oxidation. This pattern indicates that early activation of mitochondrial fatty acid oxidation and phospholipid remodelling may be part of an inflammation–driven metabolic adaptation. Increased β–oxidation and cortisone synthesis might reflect heightened energy demands and stress–axis activation, leading to mitochondrial overload and lipid–derived oxidative stress—mechanisms known to impair insulin signalling later in pregnancy (Xie et al., 2023), as well as altered acylcarnitine and sphingolipid profiles in GDM placentas (Pinto et al., 2023). Conversely, the negative correlation between IL–6 and homocysteine synthesis suggests an interaction between inflammatory signalling and one–carbon metabolism, which includes folate–and methionine–mediated transfer of one–carbon units for DNA, protein, and lipid methylation. Disruptions in this pathway may affect SAM–dependent methylation reactions, thereby impairing epigenetic and metabolic regulation of insulin signalling and lipid homeostasis in early pregnancy (Williamson et al., 2022; Jankovic-Karasoulos et al., 2021).

Leptin showed positive correlations with unsaturated phosphatidylserines and PUFA–containing phosphatidylcholines, including ether–linked species. This suggests that adipokine–driven remodelling of membrane phospholipids in early pregnancy may modify membrane fluidity and receptor function, preparing tissues for subsequent insulin resistance (Wolfgang, 2021). Likewise, positive correlations of PYY with PUFA–containing phosphatidylethanolamines, phosphatidylcholines, glycosylceramides, and bile acid metabolites imply an early role for gut–derived hormonal signalling in regulating lipid absorption, bile acid metabolism, and enterohepatic circulation—processes closely related to hepatic insulin sensitivity (Roberts et al., 2011).

**FIGURE 7:**
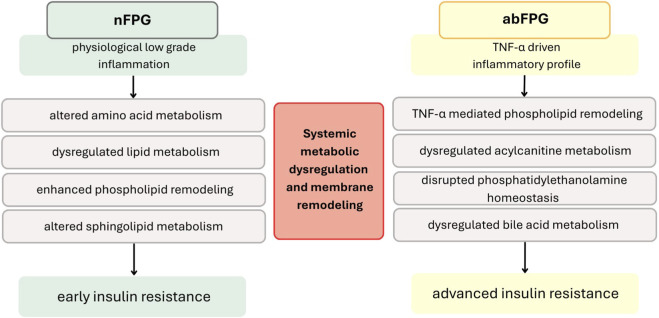
Schematic representation of coordinated interactions among inflammatory cytokines, gut hormones, and lipid metabolism pathways in early pregnancy **(A)** nFPG and **(B)** abFPG women. Colour coding: red–inflammatory cytokines (TNF-α, IL-6); orange–lipid and mitochondrial dysregulation; blue–gut hormone signaling (PYY, leptin); green–metabolic outcome (glucose homeostasis/GDM progression).

Correlations involving TNF-α, HOMA-IR, HOMA-β, and HbA1c collectively highlight early links between low–grade inflammation, insulin signalling, β–cell function, and specific membrane lipid remodelling. Notably, reviews by Pilon (2016) and Elkanawati et al. (2024) have demonstrated that, in individuals with diabetes, the cell membrane lipid composition shifts towards less fluid forms, including higher levels of saturated fatty acids, ceramides, cholesterol, and other lipid mediators, such as diacylglycerols. These changes can impair insulin signalling and β–cell function, contributing to insulin resistance and β–cell stress. Overall, these coordinated alterations likely serve as compensatory mechanisms that initially maintain normal blood glucose levels but, over time, lead to metabolic inflexibility and increased stress on β–cells (Elkanawati et al., 2024; Pilon, 2016; Alzamil, 2020). In summary, these findings suggest that in women who were metabolically healthy early in pregnancy but later developed GDM, an early, pro–inflammatory and hormonally driven shift in mitochondrial lipid metabolism, membrane composition, and bile acid signalling may establish a metabolic environment that predisposes to insulin resistance and β–cell dysfunction, ultimately leading to the development of GDM in the second trimester (see Figure 7).

In contrast, the abFPG group showed early metabolic disturbances, indicating impaired substrate utilisation and chronic low–grade inflammation. The metabolic network was characterised by pro–inflammatory and lipotoxic lipid profiles, suggesting that dysregulation of lipid metabolism may already be established in early pregnancy and predispose individuals to further metabolic decline (Pang et al., 2024). Negative correlations of IL–6 with phosphatidylserine synthase (PSS) activity, along with its positive associations with monounsaturated lysophosphatidylethanolamines (MUFA–LPEs), point to inflammation–driven phospholipid remodeling (Pang et al., 2024; Tian et al., 2023). Reduced PSS activity may limit phosphatidylserine synthesis, which is essential for cell membranes and signalling, while MUFA–LPE accumulation indicates increased phospholipid turnover and membrane stress. These alterations could compromise membrane fluidity, insulin receptor placement, and intracellular signalling pathways, thereby fostering early insulin resistance (Tian et al., 2023).

Widespread negative correlations of PYY with multiple LPE species suggest disrupted gut hormone regulation of lipid metabolism and energy balance. Since PYY normally suppresses appetite and modulates lipid absorption and oxidation, its altered association with lysophospholipids may reflect disturbed enterohepatic signalling and changed lipid partitioning, favouring hepatic steatosis and peripheral lipotoxicity (Pang et al., 2024). The correlations between TNF–α and carnitine–acylcarnitine translocase (CACT) deficiency highlight a direct link between inflammation and mitochondrial fatty acid transport. Impaired CACT function can restrict fatty acid entry into mitochondria, causing incomplete β–oxidation and buildup of acylcarnitine intermediates, metabolic markers of mitochondrial stress and insulin resistance (Xiang et al., 2025; Nicholas et al., 2019). Correlations of HOMA–IR and HOMA–BETA with changes in glycerolipid and sphingolipid pathways further emphasise early insulin resistance development, with compensatory β–cell hyperactivity. Disruptions in sphingolipid metabolism, especially ceramide accumulation, are known to impair insulin signalling, amplify inflammatory responses, and contribute to β–cell dysfunction (Glass and Olefsky, 2012).

Overall, these findings indicate that in women with impaired fasting glucose in the first trimester, inflammation–driven phospholipid remodelling, mitochondrial fatty acid transport issues, and dysregulated gut hormone signalling create a lipotoxic, pro–inflammatory metabolic environment. This combination of processes probably exacerbates insulin resistance and β–cell stress, accelerating the progression from early dysglycaemia to overt GDM in the second trimester.

One limitation of this study is the relatively small sample size and the uneven distribution of participants across study groups, which may reduce the statistical power and generalisability of the findings. Therefore, the results should be interpreted with caution. Future studies in larger, prospective cohorts are warranted to validate the observed metabolic alterations.

Additionally, this was a single-centre study conducted in a relatively homogeneous population from a specific geographic region. Although this reduces variability related to ethnicity and lifestyle, it may also limit the broader applicability of the results to other populations. Validation across multi-centre, ethnically diverse cohorts would further strengthen the clinical relevance of the findings.

## Conclusion

This study provides strong evidence that metabolomic profiling in the first trimester can detect early biochemical changes that occur before clinical signs of GDM. By analysing over 1,000 metabolites from various biochemical groups, we identified unique metabolic signatures that not only forecast GDM risk but also reveal early disturbances in glucose metabolism before the disease fully develops.

Women who were metabolically normal early in pregnancy but later developed GDM exhibited significant changes in lipid and amino acid metabolism, reflecting the progressive development of insulin resistance and β–cell stress. Conversely, those with early abnormalities in fasting glucose (abFPG) already showed a partially GDM–like metabolic profile, characterised by inflammation–driven phospholipid remodelling, ceramide accumulation, and mitochondrial dysfunction. These findings suggest that metabolic dysregulation in pregnancy follows at least two distinct pathways: one marked by a gradual failure of metabolic adaptation, and another by pre–existing impairment in substrate use and an inflammatory imbalance.

Correlation analyses further revealed that lipid–adipokine and lipid–cytokine interactions are crucial to these differing metabolic pathways. In the nFPG group, metabolite connections with leptin, IL–6, and PYY indicate a physiological response to pregnancy, while the abFPG group showed TNF–α–driven pro–inflammatory metabolic reprogramming linked to impaired lipid oxidation. Additionally, changes in amino acids, particularly increases in branched–chain amino acids and decreases in arginine and citrulline, were common across both groups, emphasising a shared mechanism involving insulin resistance and disrupted energy metabolism.

Collectively, these findings indicate that GDM is not an abrupt metabolic disturbance but rather results from early, multifactorial dysregulation of lipid, amino acid, bile acid, and inflammatory pathways. Metabolomics provides a powerful platform for early detection, risk assessment, and understanding the mechanisms behind GDM development. Future studies should aim to validate these metabolite panels in larger, multi–ethnic cohorts and integrate them with clinical and genomic data to support precision medicine approaches for GDM prevention and management.

## Data Availability

The original contributions presented in the study are included in the article/Supplementary Material, further inquiries can be directed to the corresponding author.

## Supplementary material

SUPPLEMENTARY FIGURE S1 PLS‐DA score plots illustrating the separation of the three groups: abFPG (red circles), nFPG (green circles), and NGT (blue circles), based on pareto-scaled LC-MS data.

SUPPLEMENTARY FIGURE S2 PLS‐DA plots displaying the classification of samples from groups: nFPG (green circles) and NGT (blue circles) (Pareto scaling).

SUPPLEMENTARY FIGURE S3 PLS‐DA plots showing the classification of samples from groups: abFPG (green circles) and NGT (blue circles) (Pareto scaling).

SUPPLEMENTARY FIGURE S4 Correlation analysis between clinical parameters and metabolites, grouped by metabolite class: **(a)** amino acids, **(b)** ceramides, **(c)** glycosylceramides, **(d)** phosphatidylcholines, **(e)** phosphatidylethanolamines, **(f)** phosphatidylglycerols, **(g)** phosphatidylinositols, and **(h)** metabolic processes. Spearman’s rank correlation coefficients were calculated, and *p*‐values were adjusted using the Benjamini–Hochberg (BH) correction. Significance levels are indicated as follows: p > 0.001 ***, p > 0.01 **, p > 0.05 *.

## Glossary

AAAs: aromatic amino acids
AAs: amino acids
Cers: ceramides
abGDM: abnormal fasting plasma glucose
AC: acylcarnitine
BCAAs: branched–chain amino acids
BMI: body mass index
CA: cholic acid
CACT: carnitine–carnitine–acylcarnitine translocase
CACT (NBS): carnitine–acylcarnitine translocase (newborn screening marker)
DG: diglyceride
GCTs: glucose challenge tests
GDCA: glycodeoxycholic acid
GDM: gestational diabetes mellitus
HbA1c: glycated haemoglobin
HCys: homocysteine
HDL: High–Density Lipoprotein
Hex2Cer: dihexosylceramide
HOMA–beta: Homeostatic Model Assessment of β–cell function
HOMA–IR: Homeostatic Model Assessment of Insulin Resistance
HOMA–β: homeostatic model assessment of β–cell function
IL: interleukine 6
LCFA–GluCer: long–chain fatty acid glycosylceramide
LCFA–LPS: long–chain fatty acid–lipopolysaccharide
LPE(P): lysophosphatidylethanolamine (plasmalogen form)
LPS: lipopolysaccharide
(L)PS: (lyso)phosphatidylserine
MG: monoacylglycerol
MUFA: monounsaturated fatty acid
MUFA–LPEs: monounsaturated lysophosphatidylethanolamines
nFPG: normal fasting plasma glucose
NGT: normal glucose tolerance
OC–FASM–oleoyl: containing fatty acid sphingomyelin
OGGts: oral glucose tolerance tests
PCs: phosphatidylcholines
PEs: phosphatidylethanolamines
PGs: phosphatidylglycerols
PI: phosphatidylinositol
PKU (NBS): phenylketonuria (newborn screening marker)
PLAS2: phospholipase A2
PLD: phospholipase D
Pls: plasmalogens
PSS: phosphatidylserine synthase
PUFA: polyunsaturated fatty acid
PUFA–PC: polyunsaturated fatty acid–phosphatidylcholine
PUFA–PC(O): polyunsaturated fatty acid–ether–linked phosphatidylcholine
PUFA–PE: polyunsaturated fatty acid–phosphatidylethanolamine
PUFA–PE(P): polyunsaturated fatty acid–plasmalogen phosphatidylethanolamine
PYY: peptide YY
SCAD (NBS): short–chain acyl–CoA dehydrogenase (newborn screening marker)
SFA: saturated fatty acid
SM: sphingomyelin
SO4: sulphate
SPBP d17:1: sphingoid base phosphate, d17:1
SVM: support vector machine
T2DM: type 2 diabetes mellitus
TNF alpha: Tumor Necrosis Factor alpha
UFA–PS: unsaturated fatty acid–phosphatidylserine
VLCFA: very long–chain fatty acid.
