# Supplementary figures and images for: Comprehensive first–trimester targeted metabolomics for early prediction and understanding of GDM pathophysiology
Source: Front Mol Biosci. 2026 Feb 16;13:1760710. doi: 10.3389/fmolb.2026.1760710 (PMC12950703; doi:10.3389/fmolb.2026.1760710)

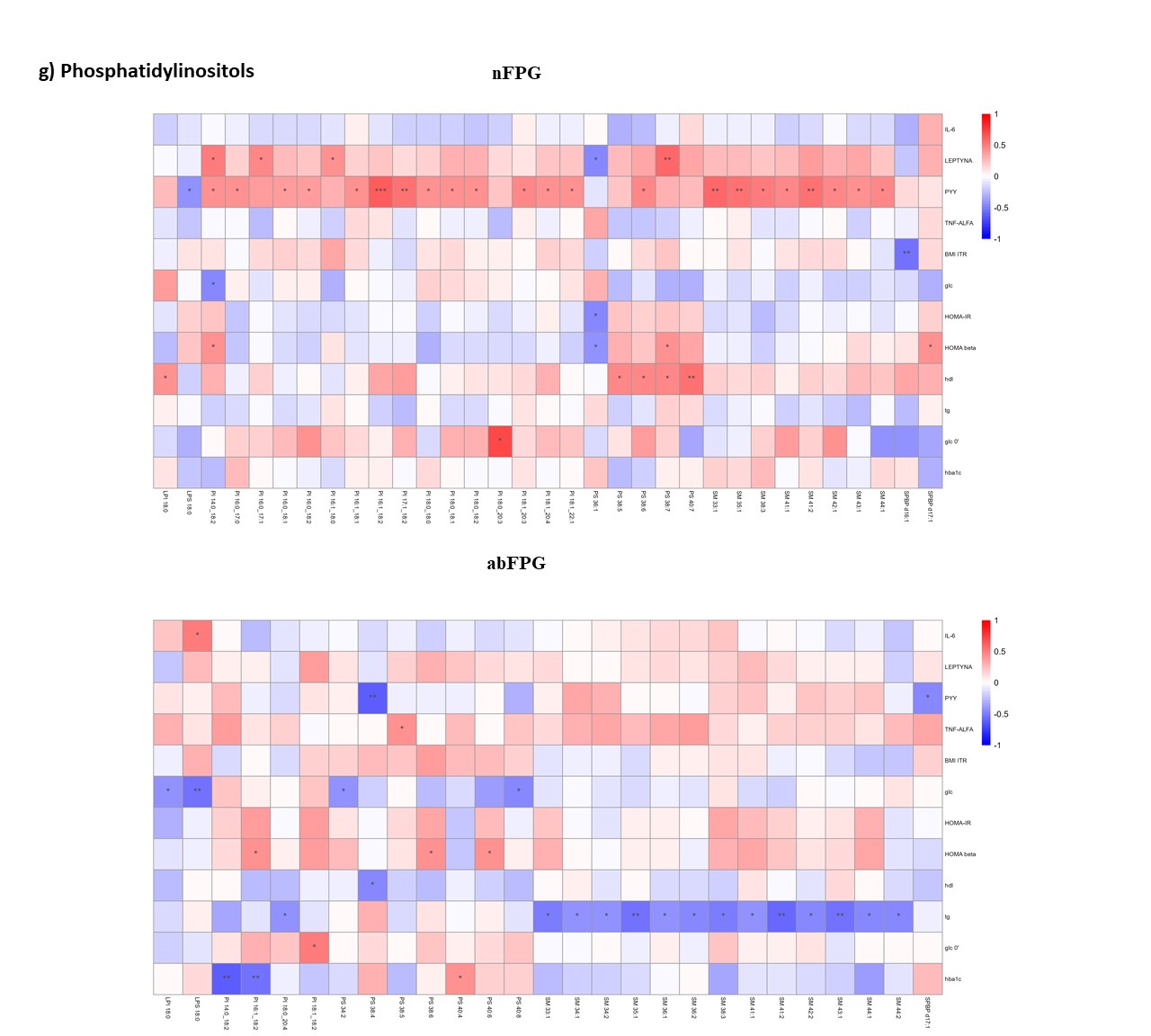

Supplement: Supplementary file 1 [file Supplementaryfile1.zip › Figure S4g.JPEG]

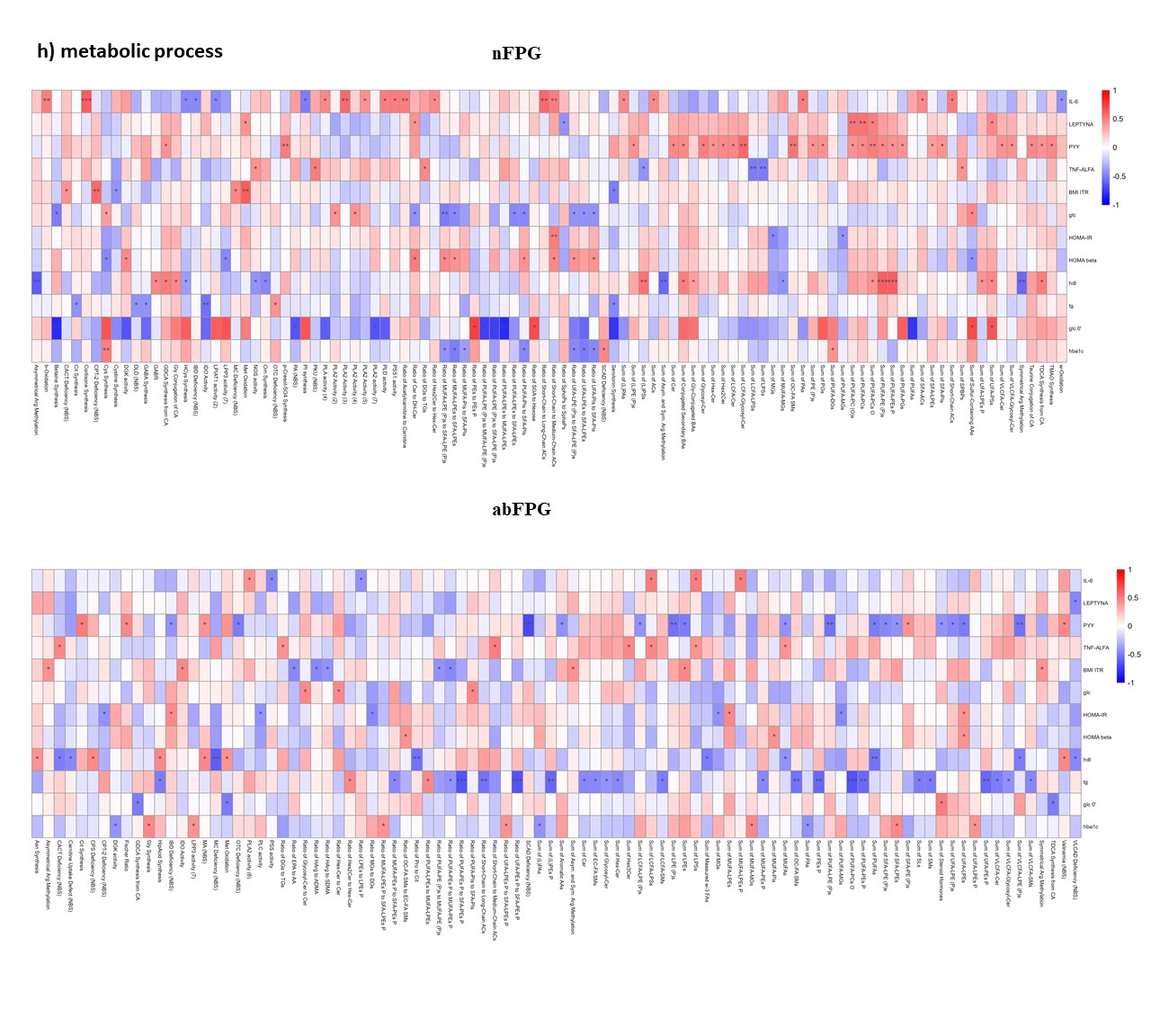

Supplement: Supplementary file 1 [file Supplementaryfile1.zip › Figure S4h.JPEG]

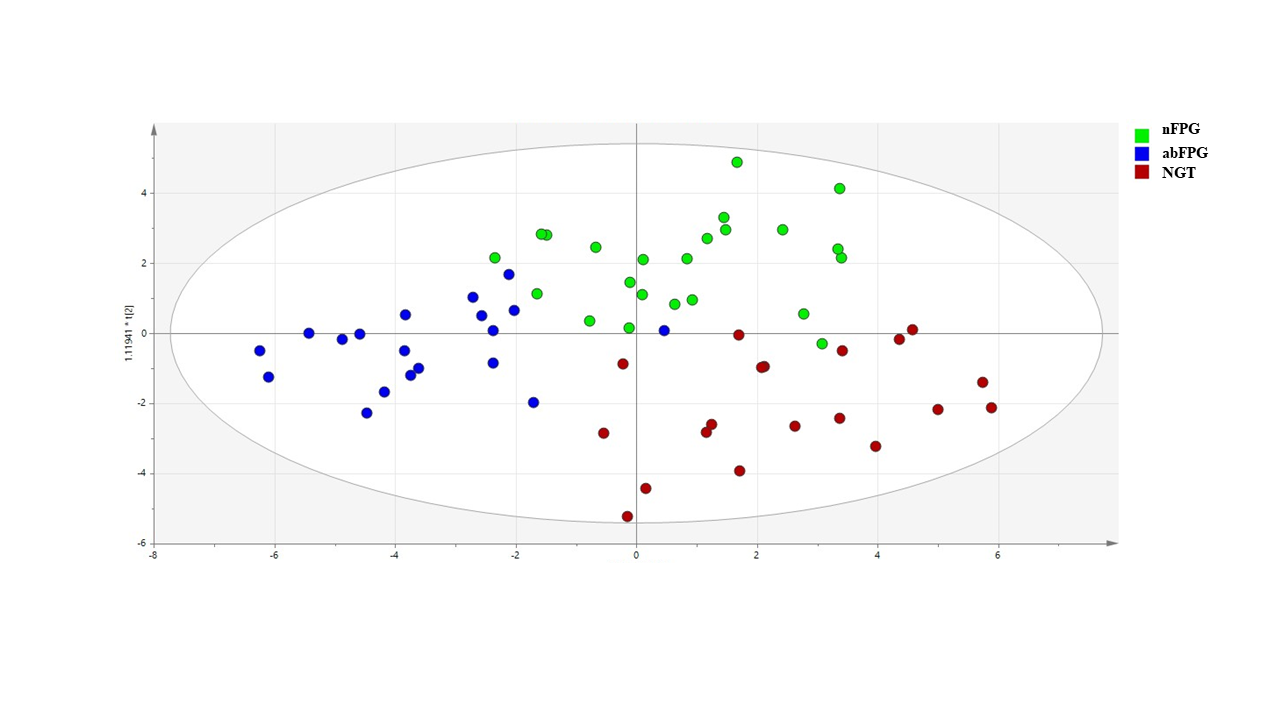

Supplement: Supplementary file 1 [file Supplementaryfile1.zip › Figure S1.TIF]

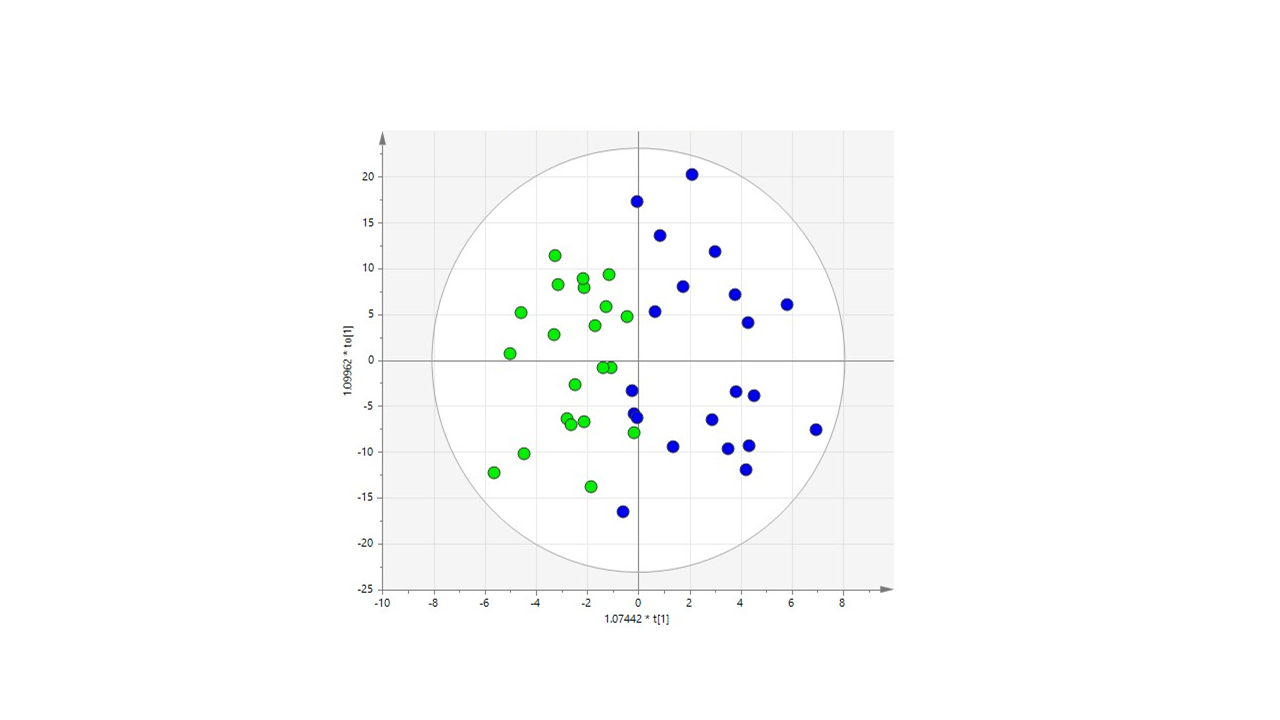

Supplement: Supplementary file 1 [file Supplementaryfile1.zip › Figure S2.TIF]

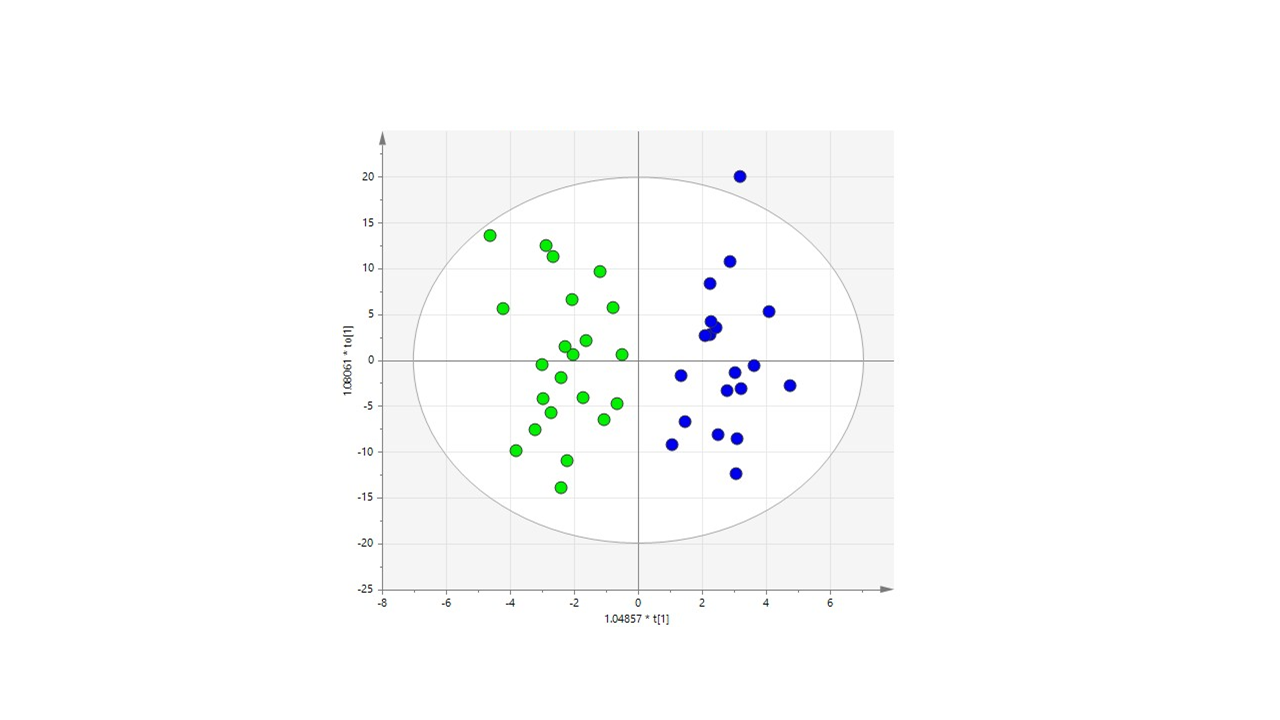

Supplement: Supplementary file 1 [file Supplementaryfile1.zip › Figure S3.TIF]

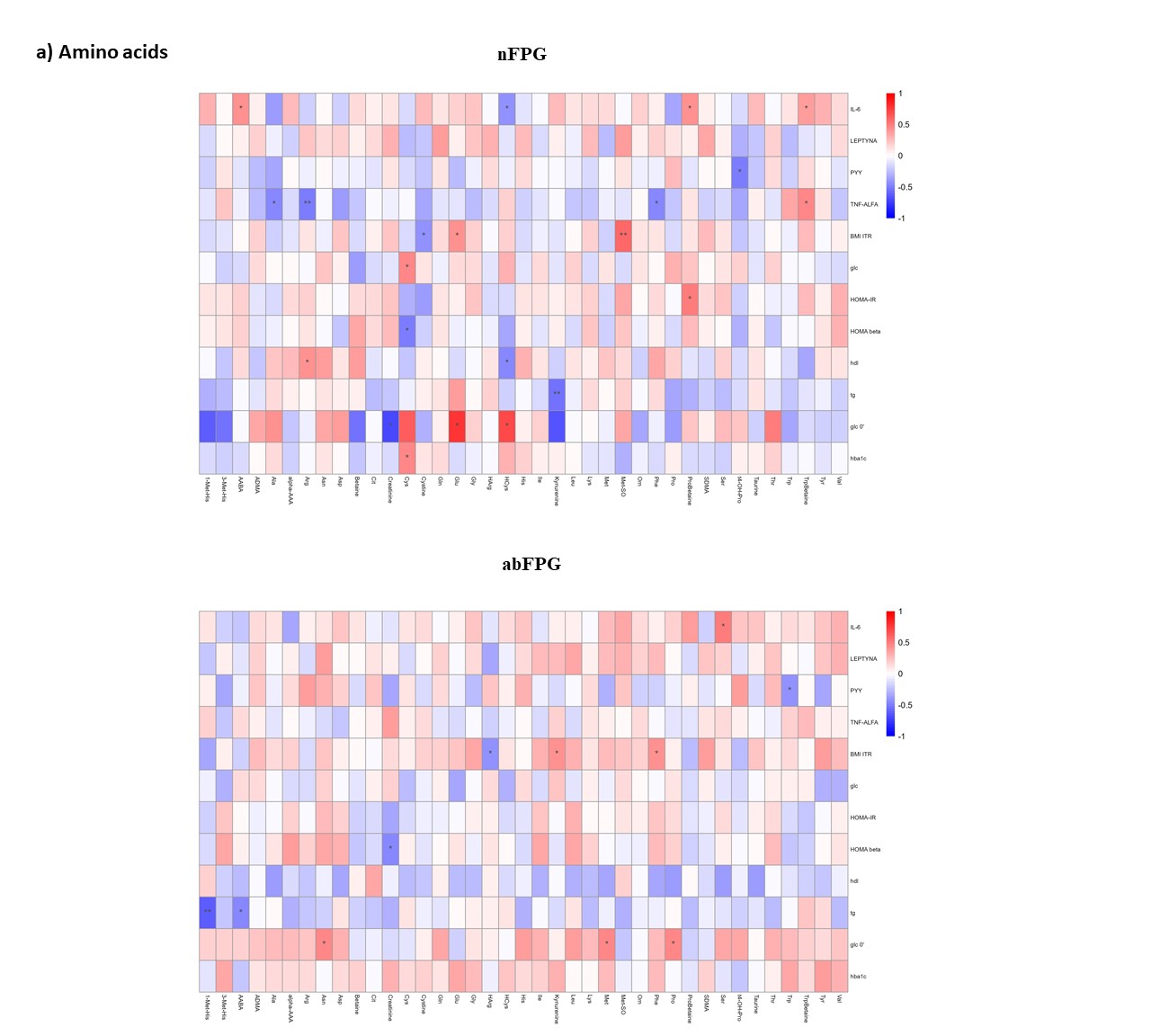

Supplement: Supplementary file 1 [file Supplementaryfile1.zip › Figure S4a.JPEG]

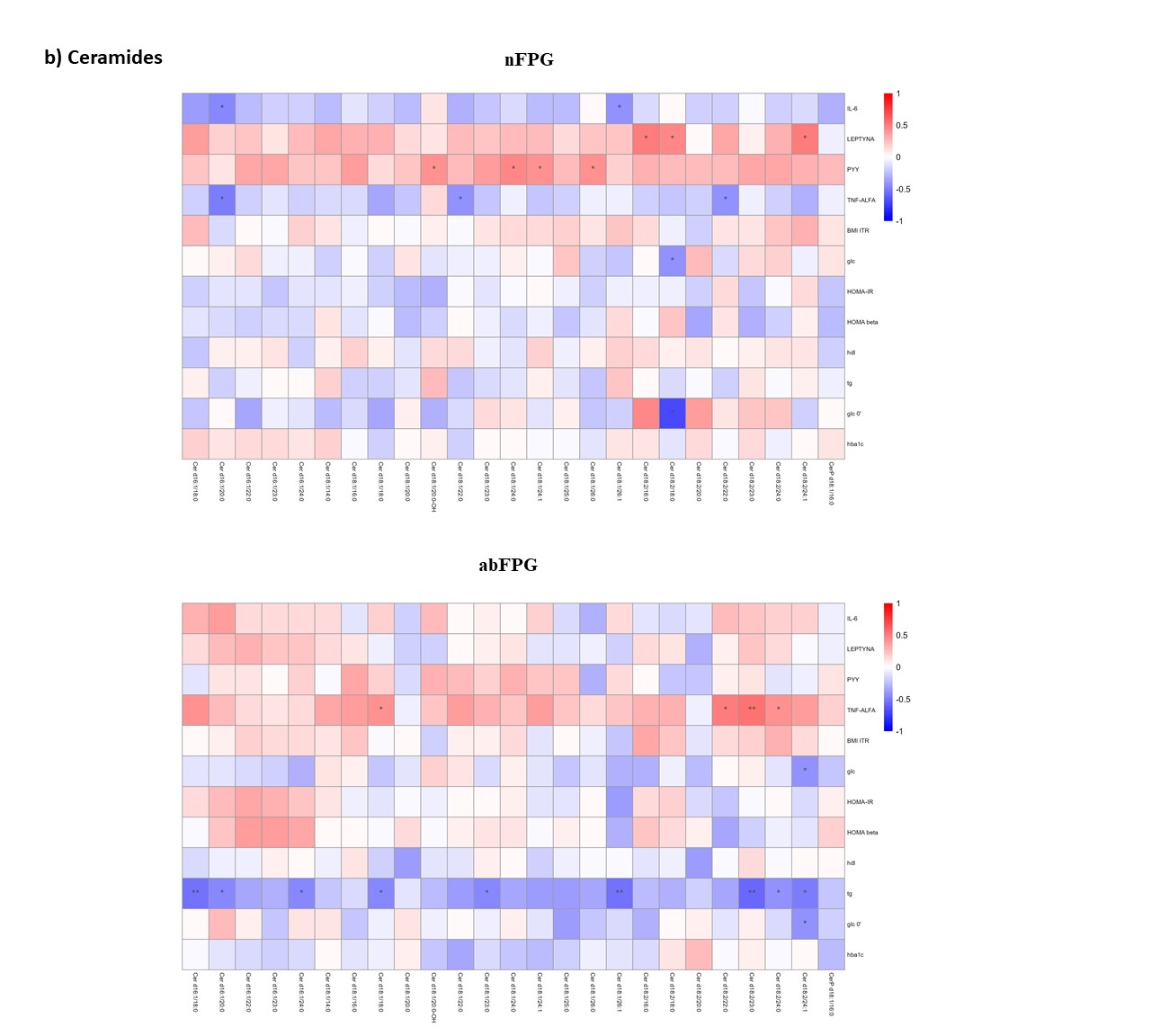

Supplement: Supplementary file 1 [file Supplementaryfile1.zip › Figure S4b.JPEG]

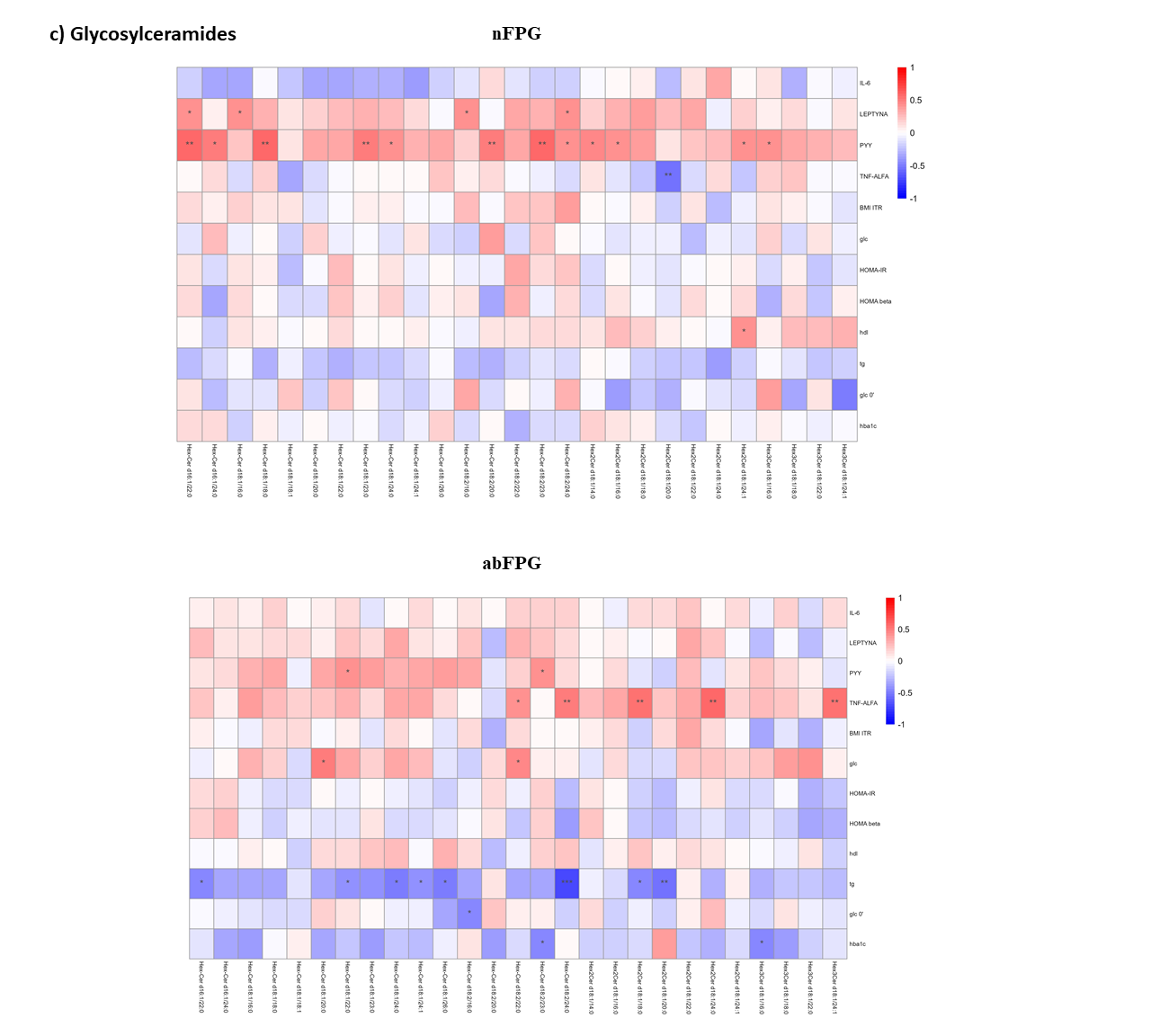

Supplement: Supplementary file 1 [file Supplementaryfile1.zip › Figure S4c.PNG]

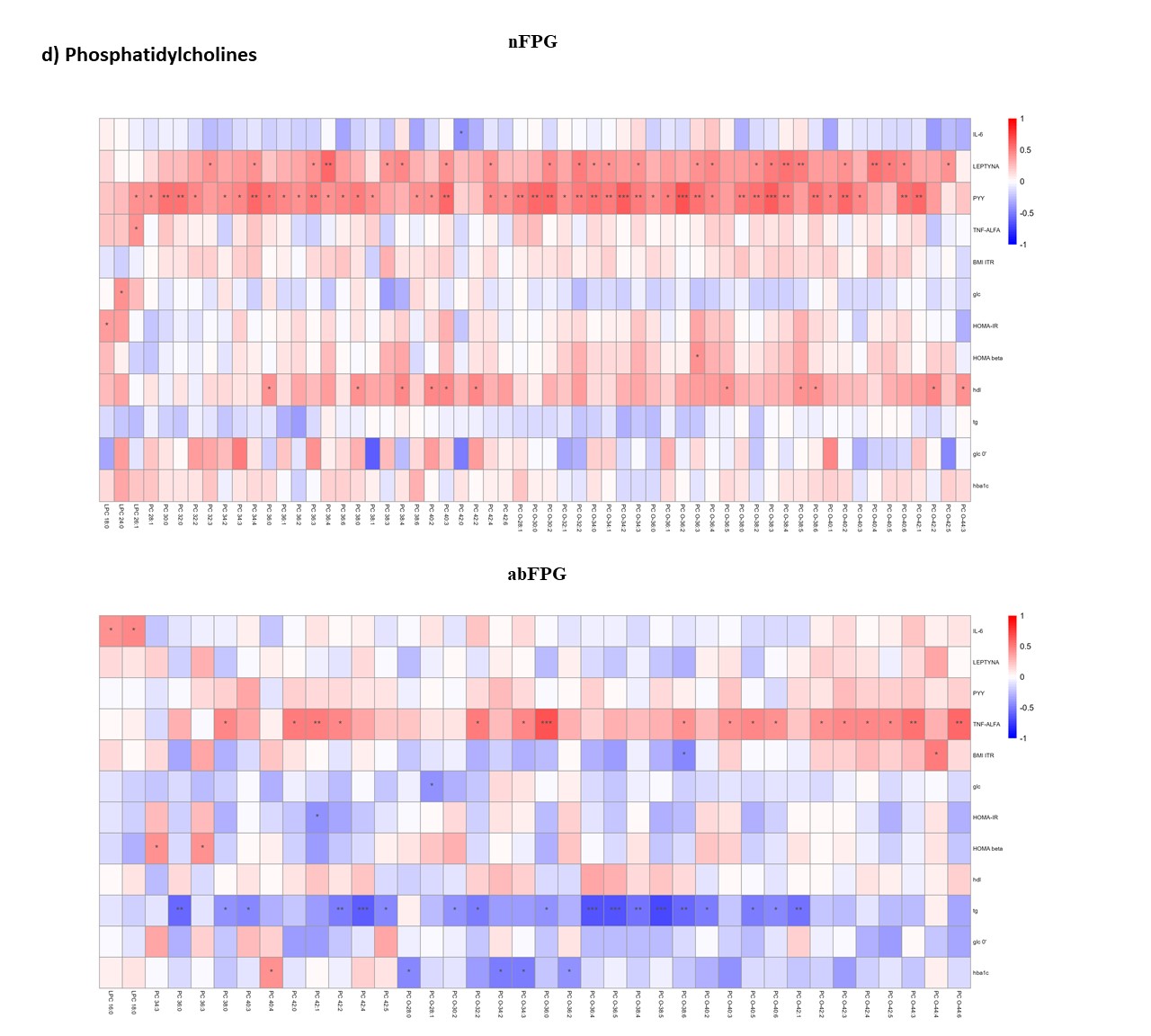

Supplement: Supplementary file 1 [file Supplementaryfile1.zip › Figure S4d.JPEG]

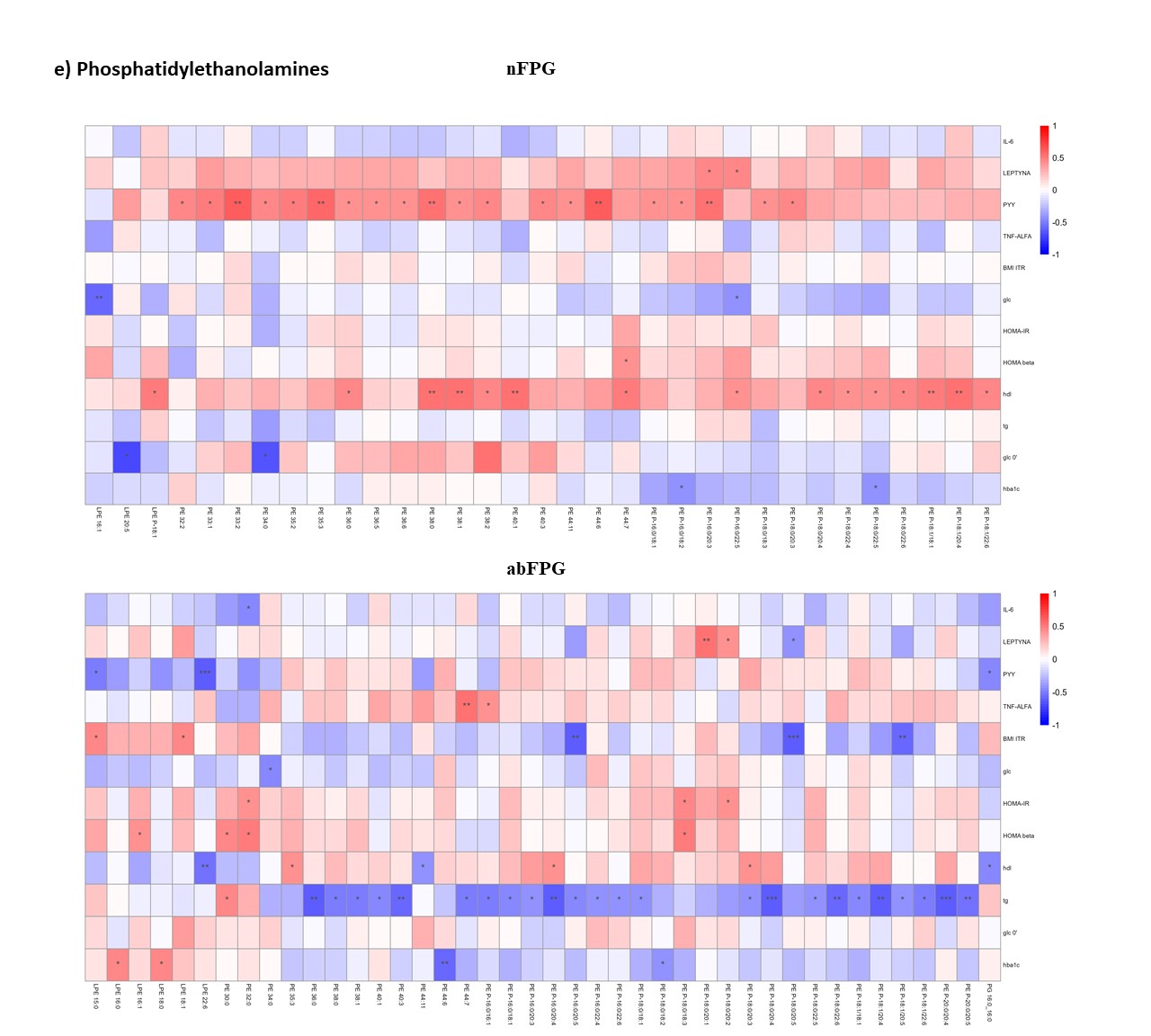

Supplement: Supplementary file 1 [file Supplementaryfile1.zip › Figure S4e.JPEG]

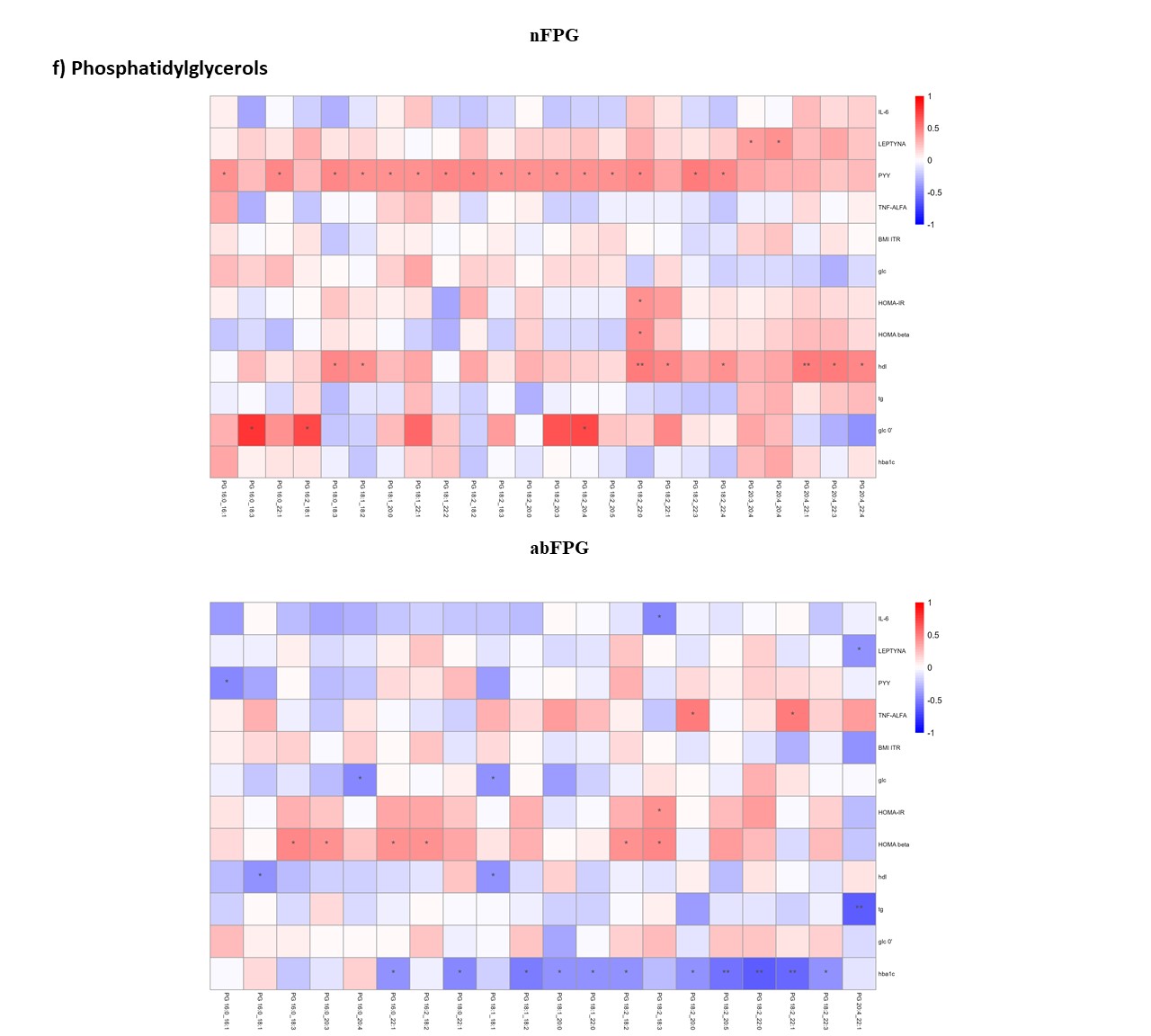

Supplement: Supplementary file 1 [file Supplementaryfile1.zip › Figure S4f.JPEG]
